# Synthesis and topology analysis of chlorido­triphen­yl(triphenyl phosphate-κ*O*)tin(IV)

**DOI:** 10.1107/S2056989023000270

**Published:** 2023-01-17

**Authors:** Serigne Fallou Pouye, Sylvain Bernès, Lamine Yaffa, Waly Diallo, Ibrahima Cissé, Cheikh Abdoul Khadir Diop, Mamadou Sidibé, Libasse Diop

**Affiliations:** a Département de Sciences Appliquées et Technologies Emergentes, Ecole Supérieure des Sciences et Techniques de l’Ingénieur, Université Amadou Mahtar Mbow, BP 45927 Dakar NAFA VDN, Dakar, Senegal; b Instituto de Física, Benemérita Universidad Autónoma de Puebla, Av. San Claudio y 18 Sur, 72570 Puebla, Pue., Mexico; c Laboratoire de Chimie Minérale et Analytique (LACHIMIA), Département de Chimie, Faculté des Sciences et Techniques, Université Cheikh Anta Diop, Dakar, Senegal; Purdue University, USA

**Keywords:** crystal structure, stannane, tri­phenyl­phosphate, QTAIM, topology analysis

## Abstract

The title Sn^IV^ complex, [Sn(C_6_H_5_)_3_Cl(C_18_H_15_O_4_P)], is a formal adduct between triphenyl phosphate (PhO)_3_P=O and the stannane derivative chlorido­tri­phenyl­tin, SnPh_3_Cl. The structure refinement reveals that this mol­ecule displays the largest Sn—O bond length for compounds including the *X*=O→SnPh_3_Cl fragment (*X* = P, S, C, or V), 2.6644 (17) Å. However, an AIM topology analysis based on the wavefunction calculated from the refined X-ray structure shows the presence of a bond critical point (3,−1), lying on the inter­basin surface separating the coordinated phosphate O atom and the Sn atom. This study thus shows that an actual polar covalent bond is formed between (PhO)_3_P=O and SnPh_3_Cl moieties.

## Abstract 2

In the title compound, a (3,−1) critical point is found on the topology path connecting the (PhO)_3_P=O and SnPh_3_Cl moieties, showing that an actual O—Sn covalent bond is formed between the phosphate and the stannane derivatives.

## 1. Chemical context

An inter­esting feature of tin(IV) is its ability to perform as a hypervalent centre: penta­coordinated tin compounds, like chlorido­(dimethyl sulfoxide)­tri­phenyl­tin, SnPh_3_(DMSO)Cl (Pouye *et al.*, 2018 ▸), are as common as tetra­coordinated tin compounds, for example chlorido­tri­phenyl­tin, SnPh_3_Cl (Tse *et al.*, 1986 ▸; Ng, 1995 ▸). This leaves the possibility open to synthesize compounds with inter­mediate valency, between four and five. The title compound is such a compound, which is formally obtained as the adduct of SnPh_3_Cl and tri­phenyl­phosphate, (PhO)_3_P=O, for which the X-ray structure is available (Svetich & Caughlan, 1965 ▸). While the phosphate group P=O coordinates the Sn centre, more than four electrons in the valence shell of Sn, 4*d* ^10^5*s* ^2^5*p* ^2^, must be involved in the formation of the bonds around Sn. Herein, we are inter­ested in the nature of the bond between Sn and the phosphate O atom. 
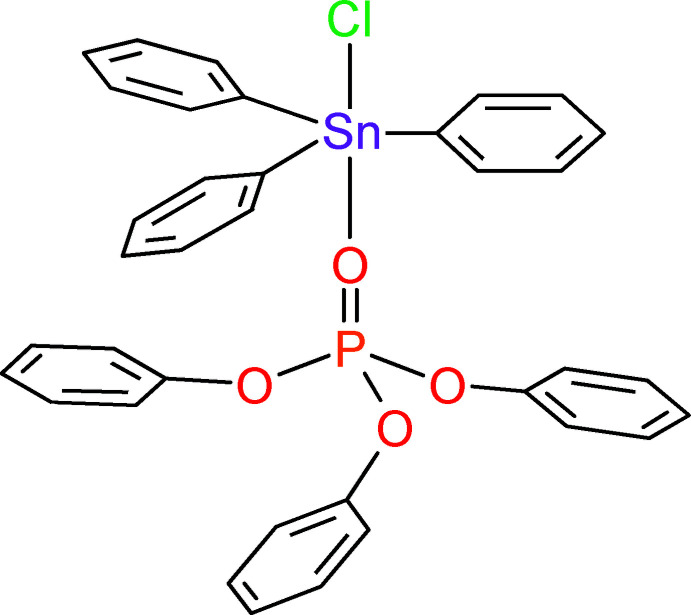


## 2. Structural commentary

**Figure 1:**
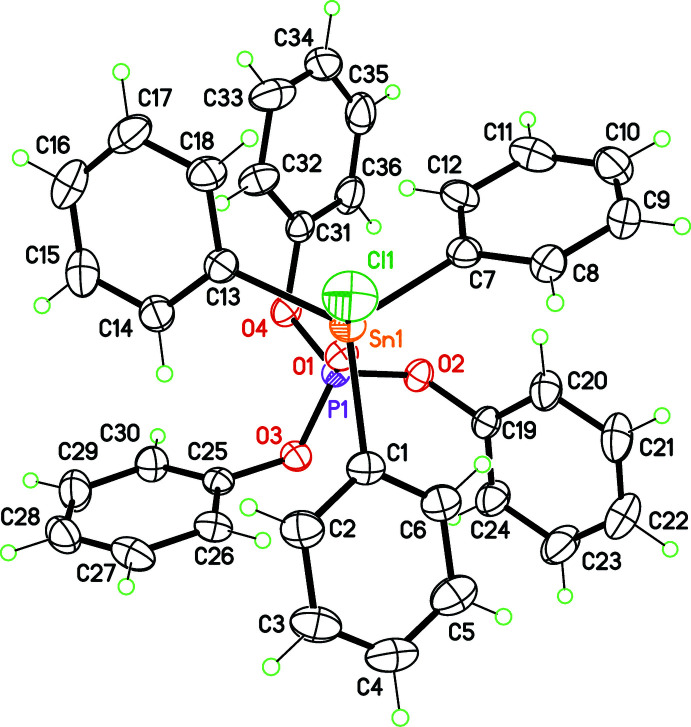
Mol­ecular structure of the title compound viewed along the P=O—Sn—Cl axis. Displacement ellipsoids for non-H atoms are drawn at the 30% probability level.

The title mol­ecule, SnPh_3_Cl-(PhO)_3_P=O, crystallizes in space group *P* 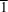 with one mol­ecule in the asymmetric unit (Fig. 1 ▸). The P=O group of the phosphate coordinates the Sn centre, *trans* to the Cl atom, with a P—O—Sn angle of 177.58 (12)°. The five-coordinate Sn centre displays a distorted trigonal–bipyramidal geometry, very different from the tetra­hedral geometry observed for SnPh_3_Cl, and consistent with *dsp* ^3^ hybrid orbitals on the metal centre. Conversely, the phosphate moiety in the title compound features a tetra­hedral geometry close to that of free (PhO)_3_P=O. The main structural feature is the staggered arrangement of the six phenyl rings, minimizing intra­molecular steric hindrance. The same conformation was previously obtained in the adduct between SnPh_3_Cl and tri­phenyl­phosphine oxide Ph_3_P=O (Ng & Kumar Das, 1992 ▸) or in the complex chlorido­[chloro­meth­yl(diphen­yl)phosphine oxide]tri­phenyl­tin, SnPh_3_Cl-Ph_2_(CH_2_Cl)P=O (Kapoor *et al.*, 2007 ▸).

In the title compound, the Sn—O bond length is 2.6644 (17) Å. A survey of the CSD shows that for *X*=O→SnPh_3_Cl fragments where *X* = P, S, C or V, the *X*=O—Sn angles range from 119.4 to 176.3°, while Sn—O bond lengths range from 2.29 to 2.64 Å (CSD 5.43 with all updates; Groom *et al.*, 2016 ▸). There is no correlation between the bond lengths and angles (*R* ^2^ = 0.002 for a linear fit). The largest Sn—O bond in the set of 40 structures retrieved from the CSD is 2.642 Å, for a dinuclear Sn complex (Gholivand *et al.*, 2015 ▸) closely related to the title compound. The title complex has thus the largest Sn—O bond length and P=O—Sn angle in this series, which could reflect a bond order less than 1 for the σ bond Sn—O. The situation is quite different, for example, for a non-hindered phosphastanninane, which forms dimers through P=O—Sn bonds, with a short Sn—O bond length of 2.425 Å (Weichmann & Meunier-Piret, 1993 ▸).

However, in the title compound, the SnPh_3_Cl moiety is certainly bound to the phosphate, since the sum of van der Waals radii for Sn and O is 3.69 Å, much larger than the observed Sn—O separation (Bondi, 1964 ▸). In other words, SnPh_3_Cl—(PhO)_3_P=O can not be described as a co-crystal between SnPh_3_Cl and (PhO)_3_P=O. This can be confirmed through the topology analysis of electron density in the complex, and in particular the computation of critical points, in the context of the Bader’s QTAIM theory (quantum theory of atoms in mol­ecules; Bader, 2009 ▸). Therefore, starting from the *SHELXL* refinement (Table 1 ▸), a wave function was calculated using *ORCA* (Neese, 2018 ▸), and the structural model further refined with *olex2.refine* and *NoSpherA2* (Bourhis *et al.*, 2015 ▸; Kleemiss *et al.*, 2021 ▸) within *OLEX2* (Dolomanov *et al.*, 2009 ▸). The relativistic basis set x2c-SVP and the generalized gradient approximation PBE functional were used. This refined model included isotropic H atoms with free coordinates, and converged to *R*_1_ = 3.26%, a slight improvement over the *SHELXL* refinement at *R*_1_ = 3.48%.

**Figure 2:**
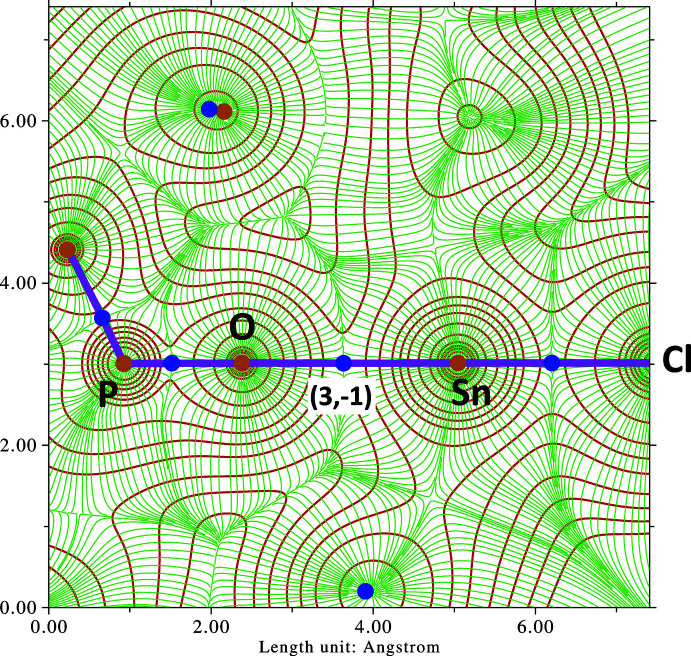
Contour map of the electron density *ρ* (brown contour lines) with the gradient vector field of *ρ* (green flux lines) in the vicinity of the P=O—Sn—Cl group. Bond and nuclear critical points are represented by blue and brown dots, respectively, while the purple bold lines are the bond paths (Bader, 2009 ▸) connecting nuclear critical points. The map was calculated and plotted using *Multiwfn* (Lu & Chen, 2012 ▸).

**Figure 3:**
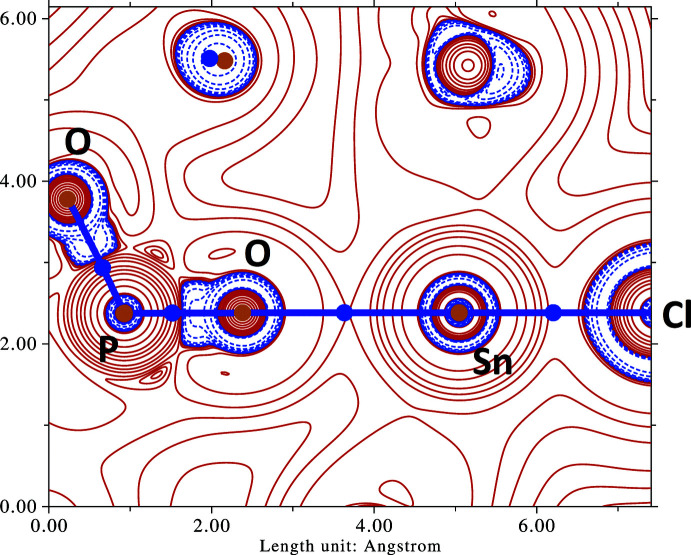
Contour map of the Laplacian of *ρ* in the vicinity of the P=O—Sn—Cl group. Solid red lines are isocontours with positive Laplacian (charge depletion regions) and dashed blue lines are isocontours with negative Laplacian (charge accumulation regions). Bond critical points and nuclear critical points are shown as blue and brown dots, respectively. The purple bold lines are the bond paths (Bader, 2009 ▸) connecting nuclear critical points in the map. The map was calculated and plotted using *Multiwfn* (Lu & Chen, 2012 ▸).

A (3,−1) bond critical point is then observed at the midpoint of the atomic pair O1/Sn1, lying on the inter­basin surface separating atoms O1 and Sn1 (Fig. 2 ▸). The charge density for this critical point is ρ = 0.024 a.u. (corresponding to 2.552 × 10 ^10^ C m^−3^), and a topology bond path connects the nuclear critical points (3,−3) placed on O1 and Sn1. The nature of the Sn1—O1 bond can be further characterized by computing the Laplacian of the electron density, 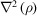 , in the vicinity of the bond: in the valence-atomic orbital region between the O and Sn atoms, the bond critical point has a small critical density and a positive Laplacian (Fig. 3 ▸). Regions combining 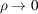 and 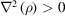 are dominated by closed-shell inter­actions suffering from Pauli repulsions, as in ionic bonds (for an extremely clear and well-written introduction to the valence-bond theory in the AIM context, see Shaik *et al.*, 2015 ▸). In the present case, the Sn1—O1 bond can thus be seen as a polar single σ (covalent) bond mainly characterized by electrostatic inter­actions. This description is obviously consistent with the large electronegativity gap between Sn and O, 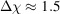 on the Pauling scale. Moreover, the bond polarization is reflected in calculated CHELPG charges (atomic charges fitting the mol­ecular electrostatic potential; Breneman & Wiberg, 1990 ▸): +0.597 for Sn1 and −0.543 for O1, as calculated by *Multiwfn* (Lu & Chen, 2012 ▸).

## 3. Supra­molecular features

**Figure 4:**
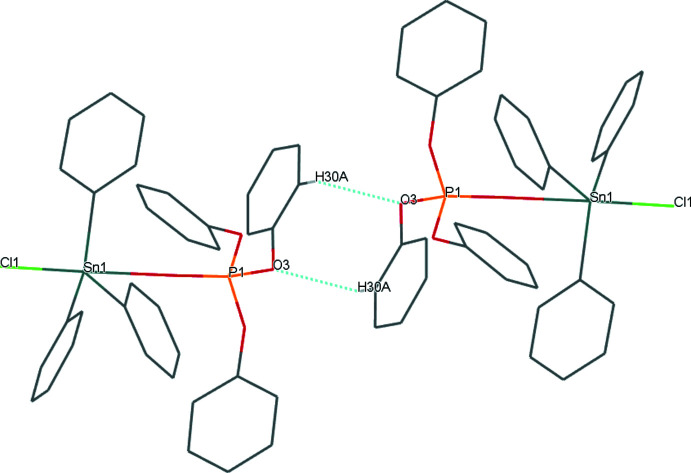
Dimeric cluster in the crystal structure, formed through weak C—H⋯O hydrogen bonds (dashed blue lines).

Although six phenyl rings are present in the mol­ecular complex, its conformation does not favour the emergence of π–π inter­actions in the crystal structure. The only relevant inter­molecular inter­actions are weak C—H⋯O contacts. Two neighbouring complexes are connected through weak inter­actions between the oxygen atoms O3 in the (PhO)_3_P=O moieties, and the hydrogen atoms H30*A* belonging to neighbouring molecules (*d*_H⋯O_ = 2.71 Å and *θ*_C—H⋯O_ = 146.8°; Table 2 ▸, entry 1). These inter­actions lead to discrete dimers, forming centrosymmetric 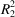 (8) ring motifs (Fig. 4 ▸). Other similar contacts in the crystal have their C—H⋯O angles below 120° (Table 2 ▸, entry 2), and are thus expected to have no contribution to crystal stabilization (Wood *et al.*, 2009 ▸).

## 4. Synthesis and crystallization

This organotin complex was synthesized by reacting Ph_3_PO_4_ (1 mmol, 326 mg) on SnPh_3_Cl (1 mmol, 385 mg) in ethanol. The mixture was refluxed (*T* = 473 K) under stirring for 1 h. The obtained solution was slightly cloudy, then it was filtered off. The filtrate was slowly evaporated at 300 K for one week, to give colourless crystals suitable for X-ray diffraction.

## 5. Refinement details

Crystal data, data collection and structure refinement details are summarized in Table 1 ▸. All H atoms were placed in calculated positions, with C—H bond lengths of 0.93 Å and *U*_iso_(H) = 1.2 *U*_eq_(carrier C atom).

## Supplementary Material

Crystal structure: contains datablock(s) I, global. DOI: 10.1107/S2056989023000270/zl4052sup1.cif

Structure factors: contains datablock(s) I. DOI: 10.1107/S2056989023000270/zl4052Isup2.hkl

CCDC reference: 2235598

Additional supporting information:  crystallographic information; 3D view; checkCIF report

## Figures and Tables

**Table 1:** Experimental details

Crystal data	
Chemical formula	[Sn(C_6_H_5_)_3_Cl(C_18_H_15_O_4_P)]
*M*_r_	711.71
Crystal system, space group	Triclinic, *P* 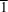
Temperature (K)	295
*a*, *b*, *c* (Å)	10.0455 (4), 12.0370 (5), 13.8304 (6)
α, β, γ (°)	93.552 (4), 93.469 (3), 93.128 (3)
*V* (Å^3^)	1663.21 (12)
*Z*	2
Radiation type	Ag *K*α, λ = 0.56083 Å
μ (mm^−1^)	0.50
Crystal size (mm)	0.40 × 0.24 × 0.16

Data collection	
Diffractometer	Stoe Stadivari
Absorption correction	Multi-scan (*X-AREA*; Stoe & Cie, 2018 ▸)
*T*_min_, *T*_max_	0.674, 1.000
No. of measured, independent and observed [*I* > 2σ(*I*)] reflections	49761, 9400, 6297
*R*_int_	0.032
(sin θ/λ)_max_ (Å^−1^)	0.697

Refinement	
*R*[*F* ^2^ > 2σ(*F* ^2^)], *wR*(*F* ^2^), *S*	0.035, 0.097, 1.00
No. of reflections	9400
No. of parameters	388
H-atom treatment	H-atom parameters constrained
Δρ_max_, Δρ_min_ (e Å^−3^)	0.76, −0.71

Computer programs: *X-AREA* (Stoe & Cie, 2018 ▸), *SHELXT2018/2* (Sheldrick, 2015*a* ▸), *SHELXL2018/3* (Sheldrick, 2015*b* ▸), *XP* in *SHELXTL-Plus* (Sheldrick, 2008 ▸) and *publCIF* (Westrip, 2010 ▸).

**Table 2:** Hydrogen-bond geometry (Å, °)

*D*—H⋯*A*	*D*—H	H⋯*A*	*D*⋯*A*	*D*—H⋯*A*
C30—H30*A*⋯O3^i^	0.93	2.71	3.526 (4)	147
C30—H30*A*⋯O2^i^	0.93	3.13	3.593 (4)	113

Symmetry code: (i) 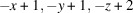 .

## supplementary crystallographic information

### Crystal data

**Table:**

[Sn(C_6_H_5_)_3_Cl(C_18_H_15_O_4_P)]	*Z* = 2
*M**_r_* = 711.71	*F*(000) = 720
Triclinic, *P*1	*D*_x_ = 1.421 Mg m^−^^3^
*a* = 10.0455 (4) Å	Ag *K*α radiation, λ = 0.56083 Å
*b* = 12.0370 (5) Å	Cell parameters from 46721 reflections
*c* = 13.8304 (6) Å	θ = 2.3–25.3°
α = 93.552 (4)°	µ = 0.50 mm^−^^1^
β = 93.469 (3)°	*T* = 295 K
γ = 93.128 (3)°	Prism, colourless
*V* = 1663.21 (12) Å^3^	0.40 × 0.24 × 0.16 mm

### Data collection

**Table:**

Stoe Stadivari diffractometer	9400 independent reflections
Radiation source: Sealed X-ray tube, Axo Astix-f Microfocus source	6297 reflections with *I* > 2σ(*I*)
Graded multilayer mirror monochromator	*R*_int_ = 0.032
Detector resolution: 5.81 pixels mm^-1^	θ_max_ = 23.0°, θ_min_ = 2.3°
ω scans	*h* = −12→13
Absorption correction: multi-scan (X-AREA; Stoe & Cie, 2018)	*k* = −16→16
*T*_min_ = 0.674, *T*_max_ = 1.000	*l* = −19→19
49761 measured reflections	

### Refinement

**Table:**

Refinement on *F*^2^	Primary atom site location: dual
Least-squares matrix: full	Secondary atom site location: difference Fourier map
*R*[*F*^2^ > 2σ(*F*^2^)] = 0.035	Hydrogen site location: inferred from neighbouring sites
*wR*(*F*^2^) = 0.097	H-atom parameters constrained
*S* = 1.00	*w* = 1/[σ^2^(*F*_o_^2^) + (0.0346*P*)^2^ + 0.9327*P*] where *P* = (*F*_o_^2^ + 2*F*_c_^2^)/3
9400 reflections	(Δ/σ)_max_ = 0.001
388 parameters	Δρ_max_ = 0.76 e Å^−^^3^
0 restraints	Δρ_min_ = −0.71 e Å^−^^3^
0 constraints	

### Fractional atomic coordinates and isotropic or equivalent isotropic displacement parameters (Å^2^)

**Table:**

	*x*	*y*	*z*	*U*_iso_*/*U*_eq_	
Sn1	0.73207 (2)	0.23155 (2)	0.60158 (2)	0.06119 (8)	
Cl1	0.79804 (12)	0.18751 (10)	0.43845 (7)	0.1046 (3)	
C1	0.8729 (3)	0.3660 (2)	0.6418 (2)	0.0604 (7)	
C2	0.8753 (4)	0.4636 (3)	0.5943 (3)	0.0792 (9)	
H2A	0.814217	0.470870	0.542093	0.095*	
C3	0.9677 (4)	0.5510 (3)	0.6234 (3)	0.0980 (12)	
H3A	0.967353	0.616668	0.591253	0.118*	
C4	1.0582 (4)	0.5410 (4)	0.6982 (4)	0.1008 (13)	
H4A	1.120050	0.599718	0.717309	0.121*	
C5	1.0591 (4)	0.4452 (4)	0.7457 (3)	0.1002 (12)	
H5A	1.122164	0.438458	0.796706	0.120*	
C6	0.9660 (3)	0.3576 (3)	0.7182 (2)	0.0774 (9)	
H6A	0.966543	0.292768	0.751420	0.093*	
C7	0.7760 (3)	0.0812 (2)	0.6660 (2)	0.0610 (7)	
C8	0.8984 (3)	0.0358 (3)	0.6565 (3)	0.0816 (9)	
H8A	0.961034	0.069823	0.619299	0.098*	
C9	0.9288 (4)	−0.0604 (4)	0.7022 (4)	0.1027 (14)	
H9A	1.011318	−0.090327	0.695210	0.123*	
C10	0.8386 (5)	−0.1106 (3)	0.7568 (4)	0.1053 (14)	
H10A	0.860158	−0.173904	0.788342	0.126*	
C11	0.7168 (5)	−0.0686 (3)	0.7656 (3)	0.0940 (11)	
H11A	0.654337	−0.104370	0.801756	0.113*	
C12	0.6851 (3)	0.0271 (3)	0.7210 (2)	0.0733 (8)	
H12A	0.601730	0.055436	0.728065	0.088*	
C13	0.5272 (3)	0.2577 (2)	0.56897 (19)	0.0601 (6)	
C14	0.4720 (3)	0.3564 (3)	0.5952 (2)	0.0714 (8)	
H14A	0.526938	0.416259	0.622712	0.086*	
C15	0.3369 (4)	0.3680 (4)	0.5813 (3)	0.0947 (12)	
H15A	0.301226	0.435244	0.599491	0.114*	
C16	0.2548 (4)	0.2808 (5)	0.5407 (3)	0.1001 (13)	
H16A	0.163297	0.288594	0.532147	0.120*	
C17	0.3069 (4)	0.1832 (4)	0.5131 (3)	0.0960 (12)	
H17A	0.250940	0.124019	0.485647	0.115*	
C18	0.4433 (3)	0.1712 (3)	0.5257 (2)	0.0771 (9)	
H18A	0.478618	0.104607	0.504898	0.093*	
P1	0.61706 (6)	0.31982 (6)	0.87530 (5)	0.04891 (15)	
O1	0.65797 (17)	0.28553 (15)	0.77937 (12)	0.0551 (4)	
O2	0.69211 (17)	0.26752 (17)	0.96296 (13)	0.0599 (4)	
O3	0.63487 (19)	0.44813 (15)	0.90462 (13)	0.0581 (4)	
O4	0.46665 (17)	0.29271 (16)	0.89412 (14)	0.0599 (5)	
C19	0.8332 (3)	0.2685 (2)	0.97307 (19)	0.0580 (6)	
C20	0.8990 (3)	0.1840 (3)	0.9319 (2)	0.0817 (10)	
H20A	0.852379	0.127052	0.893273	0.098*	
C21	1.0357 (4)	0.1836 (4)	0.9480 (3)	0.1044 (14)	
H21A	1.081703	0.124937	0.922090	0.125*	
C22	1.1033 (4)	0.2699 (5)	1.0023 (3)	0.1053 (14)	
H22A	1.195691	0.270597	1.012357	0.126*	
C23	1.0363 (4)	0.3540 (4)	1.0413 (3)	0.1084 (14)	
H23A	1.083077	0.412464	1.078131	0.130*	
C24	0.8999 (3)	0.3543 (3)	1.0273 (3)	0.0854 (10)	
H24A	0.853900	0.412281	1.054415	0.102*	
C25	0.5859 (3)	0.5270 (2)	0.84188 (18)	0.0531 (6)	
C26	0.6648 (3)	0.5650 (2)	0.7723 (2)	0.0670 (7)	
H26A	0.749342	0.538655	0.765506	0.080*	
C27	0.6166 (4)	0.6431 (3)	0.7122 (3)	0.0854 (10)	
H27A	0.668221	0.668757	0.663711	0.103*	
C28	0.4931 (5)	0.6831 (3)	0.7239 (3)	0.0926 (12)	
H28A	0.460557	0.735260	0.683014	0.111*	
C29	0.4173 (4)	0.6455 (3)	0.7964 (3)	0.0966 (12)	
H29A	0.334446	0.674085	0.805287	0.116*	
C30	0.4631 (3)	0.5663 (3)	0.8557 (2)	0.0732 (8)	
H30A	0.411566	0.540112	0.904119	0.088*	
C31	0.4067 (2)	0.1843 (2)	0.8716 (2)	0.0599 (7)	
C32	0.3518 (3)	0.1565 (3)	0.7802 (3)	0.0808 (9)	
H32A	0.355169	0.207309	0.732295	0.097*	
C33	0.2910 (4)	0.0513 (4)	0.7604 (4)	0.1065 (14)	
H33A	0.254556	0.030130	0.698124	0.128*	
C34	0.2843 (4)	−0.0213 (4)	0.8311 (5)	0.126 (2)	
H34A	0.243098	−0.091981	0.817172	0.151*	
C35	0.3373 (4)	0.0085 (4)	0.9224 (5)	0.133 (2)	
H35A	0.331360	−0.041837	0.970579	0.159*	
C36	0.4003 (3)	0.1133 (3)	0.9443 (3)	0.0913 (12)	
H36A	0.436863	0.134381	1.006540	0.110*	

### Atomic displacement parameters (Å^2^)

**Table:**

	*U*^11^	*U*^22^	*U*^33^	*U*^12^	*U*^13^	*U*^23^
Sn1	0.06419 (12)	0.06082 (13)	0.05806 (12)	0.00085 (9)	0.00682 (8)	−0.00058 (8)
Cl1	0.1275 (8)	0.1191 (8)	0.0673 (5)	0.0055 (7)	0.0309 (5)	−0.0156 (5)
C1	0.0594 (15)	0.0638 (17)	0.0582 (16)	−0.0030 (13)	0.0183 (12)	−0.0024 (13)
C2	0.084 (2)	0.083 (2)	0.072 (2)	−0.0097 (18)	0.0163 (17)	0.0134 (17)
C3	0.107 (3)	0.084 (3)	0.105 (3)	−0.025 (2)	0.030 (2)	0.017 (2)
C4	0.091 (3)	0.095 (3)	0.112 (3)	−0.033 (2)	0.024 (2)	−0.016 (3)
C5	0.086 (2)	0.116 (3)	0.092 (3)	−0.014 (2)	−0.009 (2)	−0.014 (2)
C6	0.0771 (19)	0.078 (2)	0.075 (2)	−0.0028 (17)	0.0031 (16)	−0.0006 (17)
C7	0.0608 (15)	0.0557 (16)	0.0642 (17)	0.0002 (12)	0.0015 (13)	−0.0097 (13)
C8	0.0617 (17)	0.077 (2)	0.105 (3)	0.0056 (16)	−0.0001 (17)	−0.0011 (19)
C9	0.076 (2)	0.085 (3)	0.144 (4)	0.019 (2)	−0.029 (2)	0.000 (3)
C10	0.124 (4)	0.067 (2)	0.121 (4)	0.008 (2)	−0.030 (3)	0.011 (2)
C11	0.129 (3)	0.065 (2)	0.087 (3)	−0.014 (2)	0.016 (2)	0.0034 (18)
C12	0.082 (2)	0.0543 (17)	0.083 (2)	0.0007 (15)	0.0145 (17)	−0.0064 (15)
C13	0.0697 (16)	0.0635 (17)	0.0458 (14)	0.0025 (13)	−0.0029 (12)	0.0015 (12)
C14	0.084 (2)	0.0667 (19)	0.0622 (18)	0.0093 (16)	−0.0065 (15)	−0.0029 (14)
C15	0.099 (3)	0.108 (3)	0.080 (2)	0.043 (2)	−0.003 (2)	0.005 (2)
C16	0.070 (2)	0.153 (4)	0.078 (2)	0.017 (3)	−0.0119 (18)	0.016 (3)
C17	0.082 (2)	0.120 (3)	0.080 (2)	−0.022 (2)	−0.0217 (19)	0.008 (2)
C18	0.089 (2)	0.071 (2)	0.0669 (19)	−0.0022 (17)	−0.0087 (16)	−0.0092 (16)
P1	0.0494 (3)	0.0549 (4)	0.0420 (3)	0.0027 (3)	0.0035 (3)	−0.0006 (3)
O1	0.0571 (9)	0.0637 (11)	0.0437 (9)	0.0015 (8)	0.0061 (7)	−0.0044 (8)
O2	0.0549 (10)	0.0743 (13)	0.0510 (10)	0.0046 (9)	0.0009 (8)	0.0111 (9)
O3	0.0720 (11)	0.0541 (10)	0.0464 (9)	0.0041 (9)	−0.0033 (8)	−0.0042 (8)
O4	0.0513 (9)	0.0674 (12)	0.0615 (11)	0.0064 (9)	0.0094 (8)	0.0008 (9)
C19	0.0562 (14)	0.0694 (18)	0.0484 (14)	0.0061 (13)	−0.0022 (11)	0.0069 (12)
C20	0.080 (2)	0.093 (2)	0.070 (2)	0.0265 (18)	−0.0112 (16)	−0.0145 (18)
C21	0.088 (3)	0.128 (4)	0.100 (3)	0.053 (3)	0.000 (2)	−0.005 (3)
C22	0.063 (2)	0.155 (4)	0.097 (3)	0.016 (2)	−0.011 (2)	0.005 (3)
C23	0.073 (2)	0.128 (4)	0.117 (3)	−0.002 (2)	−0.020 (2)	−0.019 (3)
C24	0.074 (2)	0.091 (2)	0.087 (2)	0.0094 (18)	−0.0114 (18)	−0.0196 (19)
C25	0.0626 (14)	0.0483 (14)	0.0464 (13)	−0.0016 (11)	0.0005 (11)	−0.0056 (11)
C26	0.0746 (18)	0.0590 (17)	0.0669 (18)	−0.0069 (14)	0.0164 (14)	−0.0012 (14)
C27	0.126 (3)	0.0600 (19)	0.070 (2)	−0.012 (2)	0.015 (2)	0.0070 (16)
C28	0.126 (3)	0.066 (2)	0.083 (3)	0.004 (2)	−0.022 (2)	0.0166 (18)
C29	0.085 (2)	0.096 (3)	0.112 (3)	0.028 (2)	−0.001 (2)	0.018 (2)
C30	0.0692 (18)	0.081 (2)	0.071 (2)	0.0089 (16)	0.0116 (15)	0.0089 (16)
C31	0.0414 (12)	0.0672 (17)	0.0730 (18)	0.0040 (12)	0.0091 (12)	0.0137 (14)
C32	0.0707 (19)	0.091 (2)	0.078 (2)	−0.0193 (17)	0.0099 (16)	0.0018 (18)
C33	0.078 (2)	0.108 (3)	0.127 (4)	−0.028 (2)	0.010 (2)	−0.022 (3)
C34	0.069 (2)	0.075 (3)	0.232 (7)	−0.010 (2)	−0.009 (3)	0.016 (4)
C35	0.071 (2)	0.109 (4)	0.224 (6)	−0.005 (2)	−0.017 (3)	0.096 (4)
C36	0.0629 (18)	0.107 (3)	0.107 (3)	0.0014 (18)	−0.0116 (18)	0.050 (2)

### Geometric parameters (Å, º)

**Table:**

Sn1—C1	2.116 (3)	P1—O4	1.5690 (18)
Sn1—C7	2.122 (3)	P1—O3	1.5701 (19)
Sn1—C13	2.124 (3)	P1—O2	1.5718 (19)
Sn1—Cl1	2.4252 (9)	O2—C19	1.415 (3)
Sn1—O1	2.6644 (17)	O3—C25	1.414 (3)
C1—C6	1.380 (4)	O4—C31	1.416 (3)
C1—C2	1.381 (4)	C19—C20	1.357 (4)
C2—C3	1.388 (5)	C19—C24	1.359 (4)
C2—H2A	0.9300	C20—C21	1.378 (5)
C3—C4	1.351 (6)	C20—H20A	0.9300
C3—H3A	0.9300	C21—C22	1.367 (6)
C4—C5	1.362 (6)	C21—H21A	0.9300
C4—H4A	0.9300	C22—C23	1.349 (6)
C5—C6	1.389 (5)	C22—H22A	0.9300
C5—H5A	0.9300	C23—C24	1.373 (5)
C6—H6A	0.9300	C23—H23A	0.9300
C7—C8	1.382 (4)	C24—H24A	0.9300
C7—C12	1.385 (4)	C25—C30	1.365 (4)
C8—C9	1.393 (5)	C25—C26	1.368 (4)
C8—H8A	0.9300	C26—C27	1.381 (5)
C9—C10	1.355 (6)	C26—H26A	0.9300
C9—H9A	0.9300	C27—C28	1.369 (6)
C10—C11	1.359 (6)	C27—H27A	0.9300
C10—H10A	0.9300	C28—C29	1.379 (6)
C11—C12	1.382 (5)	C28—H28A	0.9300
C11—H11A	0.9300	C29—C30	1.377 (5)
C12—H12A	0.9300	C29—H29A	0.9300
C13—C14	1.376 (4)	C30—H30A	0.9300
C13—C18	1.386 (4)	C31—C36	1.362 (4)
C14—C15	1.375 (5)	C31—C32	1.362 (4)
C14—H14A	0.9300	C32—C33	1.380 (5)
C15—C16	1.368 (6)	C32—H32A	0.9300
C15—H15A	0.9300	C33—C34	1.354 (7)
C16—C17	1.355 (6)	C33—H33A	0.9300
C16—H16A	0.9300	C34—C35	1.359 (7)
C17—C18	1.387 (5)	C34—H34A	0.9300
C17—H17A	0.9300	C35—C36	1.388 (6)
C18—H18A	0.9300	C35—H35A	0.9300
P1—O1	1.4547 (18)	C36—H36A	0.9300
			
C1—Sn1—C7	114.01 (11)	O4—P1—O3	102.20 (11)
C1—Sn1—C13	121.56 (11)	O1—P1—O2	115.88 (11)
C7—Sn1—C13	116.86 (11)	O4—P1—O2	102.24 (10)
C1—Sn1—Cl1	98.73 (8)	O3—P1—O2	102.52 (10)
C7—Sn1—Cl1	99.82 (8)	P1—O1—Sn1	177.58 (12)
C13—Sn1—Cl1	99.13 (8)	C19—O2—P1	121.84 (16)
C6—C1—C2	118.1 (3)	C25—O3—P1	120.89 (15)
C6—C1—Sn1	119.7 (2)	C31—O4—P1	120.63 (16)
C2—C1—Sn1	122.2 (2)	C20—C19—C24	121.3 (3)
C1—C2—C3	120.8 (4)	C20—C19—O2	120.6 (3)
C1—C2—H2A	119.6	C24—C19—O2	118.1 (3)
C3—C2—H2A	119.6	C19—C20—C21	119.3 (4)
C4—C3—C2	120.2 (4)	C19—C20—H20A	120.3
C4—C3—H3A	119.9	C21—C20—H20A	120.3
C2—C3—H3A	119.9	C22—C21—C20	119.6 (4)
C3—C4—C5	120.2 (4)	C22—C21—H21A	120.2
C3—C4—H4A	119.9	C20—C21—H21A	120.2
C5—C4—H4A	119.9	C23—C22—C21	120.2 (4)
C4—C5—C6	120.2 (4)	C23—C22—H22A	119.9
C4—C5—H5A	119.9	C21—C22—H22A	119.9
C6—C5—H5A	119.9	C22—C23—C24	120.7 (4)
C1—C6—C5	120.5 (4)	C22—C23—H23A	119.7
C1—C6—H6A	119.8	C24—C23—H23A	119.7
C5—C6—H6A	119.8	C19—C24—C23	118.9 (4)
C8—C7—C12	117.8 (3)	C19—C24—H24A	120.6
C8—C7—Sn1	120.8 (2)	C23—C24—H24A	120.6
C12—C7—Sn1	121.4 (2)	C30—C25—C26	122.2 (3)
C7—C8—C9	120.6 (4)	C30—C25—O3	118.5 (2)
C7—C8—H8A	119.7	C26—C25—O3	119.2 (3)
C9—C8—H8A	119.7	C25—C26—C27	118.7 (3)
C10—C9—C8	120.3 (4)	C25—C26—H26A	120.7
C10—C9—H9A	119.8	C27—C26—H26A	120.7
C8—C9—H9A	119.8	C28—C27—C26	120.3 (3)
C9—C10—C11	120.0 (4)	C28—C27—H27A	119.8
C9—C10—H10A	120.0	C26—C27—H27A	119.8
C11—C10—H10A	120.0	C27—C28—C29	119.7 (3)
C10—C11—C12	120.4 (4)	C27—C28—H28A	120.2
C10—C11—H11A	119.8	C29—C28—H28A	120.2
C12—C11—H11A	119.8	C30—C29—C28	120.7 (4)
C11—C12—C7	120.8 (3)	C30—C29—H29A	119.7
C11—C12—H12A	119.6	C28—C29—H29A	119.7
C7—C12—H12A	119.6	C25—C30—C29	118.4 (3)
C14—C13—C18	118.1 (3)	C25—C30—H30A	120.8
C14—C13—Sn1	122.1 (2)	C29—C30—H30A	120.8
C18—C13—Sn1	119.7 (2)	C36—C31—C32	122.4 (3)
C15—C14—C13	121.0 (3)	C36—C31—O4	118.1 (3)
C15—C14—H14A	119.5	C32—C31—O4	119.4 (3)
C13—C14—H14A	119.5	C31—C32—C33	118.5 (4)
C16—C15—C14	120.2 (4)	C31—C32—H32A	120.8
C16—C15—H15A	119.9	C33—C32—H32A	120.8
C14—C15—H15A	119.9	C34—C33—C32	120.3 (4)
C17—C16—C15	119.9 (4)	C34—C33—H33A	119.9
C17—C16—H16A	120.0	C32—C33—H33A	119.9
C15—C16—H16A	120.0	C33—C34—C35	120.5 (4)
C16—C17—C18	120.3 (4)	C33—C34—H34A	119.7
C16—C17—H17A	119.8	C35—C34—H34A	119.7
C18—C17—H17A	119.8	C34—C35—C36	120.5 (4)
C13—C18—C17	120.4 (4)	C34—C35—H35A	119.7
C13—C18—H18A	119.8	C36—C35—H35A	119.7
C17—C18—H18A	119.8	C31—C36—C35	117.7 (4)
O1—P1—O4	116.16 (11)	C31—C36—H36A	121.1
O1—P1—O3	115.72 (11)	C35—C36—H36A	121.1
			
C6—C1—C2—C3	−0.8 (5)	O2—P1—O4—C31	−77.4 (2)
Sn1—C1—C2—C3	179.0 (3)	P1—O2—C19—C20	−89.0 (3)
C1—C2—C3—C4	0.9 (6)	P1—O2—C19—C24	92.9 (3)
C2—C3—C4—C5	−0.2 (7)	C24—C19—C20—C21	2.1 (5)
C3—C4—C5—C6	−0.7 (7)	O2—C19—C20—C21	−176.0 (3)
C2—C1—C6—C5	0.0 (5)	C19—C20—C21—C22	−2.2 (6)
Sn1—C1—C6—C5	−179.9 (3)	C20—C21—C22—C23	1.2 (7)
C4—C5—C6—C1	0.8 (6)	C21—C22—C23—C24	0.0 (8)
C12—C7—C8—C9	−0.8 (5)	C20—C19—C24—C23	−0.9 (6)
Sn1—C7—C8—C9	177.4 (3)	O2—C19—C24—C23	177.2 (3)
C7—C8—C9—C10	−0.2 (6)	C22—C23—C24—C19	−0.2 (7)
C8—C9—C10—C11	1.4 (7)	P1—O3—C25—C30	95.6 (3)
C9—C10—C11—C12	−1.6 (7)	P1—O3—C25—C26	−87.1 (3)
C10—C11—C12—C7	0.6 (6)	C30—C25—C26—C27	−2.0 (4)
C8—C7—C12—C11	0.6 (5)	O3—C25—C26—C27	−179.2 (3)
Sn1—C7—C12—C11	−177.6 (3)	C25—C26—C27—C28	1.3 (5)
C18—C13—C14—C15	1.7 (5)	C26—C27—C28—C29	0.5 (6)
Sn1—C13—C14—C15	−174.0 (3)	C27—C28—C29—C30	−1.6 (6)
C13—C14—C15—C16	−0.1 (6)	C26—C25—C30—C29	0.9 (5)
C14—C15—C16—C17	−0.8 (6)	O3—C25—C30—C29	178.1 (3)
C15—C16—C17—C18	−0.1 (6)	C28—C29—C30—C25	0.9 (6)
C14—C13—C18—C17	−2.6 (5)	P1—O4—C31—C36	96.4 (3)
Sn1—C13—C18—C17	173.3 (3)	P1—O4—C31—C32	−86.9 (3)
C16—C17—C18—C13	1.8 (6)	C36—C31—C32—C33	−2.0 (5)
O1—P1—O2—C19	49.9 (2)	O4—C31—C32—C33	−178.5 (3)
O4—P1—O2—C19	177.3 (2)	C31—C32—C33—C34	1.3 (6)
O3—P1—O2—C19	−77.1 (2)	C32—C33—C34—C35	−0.1 (7)
O1—P1—O3—C25	50.1 (2)	C33—C34—C35—C36	−0.6 (8)
O4—P1—O3—C25	−77.1 (2)	C32—C31—C36—C35	1.3 (5)
O2—P1—O3—C25	177.17 (18)	O4—C31—C36—C35	177.8 (3)
O1—P1—O4—C31	49.7 (2)	C34—C35—C36—C31	0.0 (7)
O3—P1—O4—C31	176.67 (19)		

### Hydrogen-bond geometry (Å, º)

**Table:**

*D*—H···*A*	*D*—H	H···*A*	*D*···*A*	*D*—H···*A*
C30—H30*A*···O3^i^	0.93	2.71	3.526 (4)	147
C30—H30*A*···O2^i^	0.93	3.13	3.593 (4)	113

Symmetry code: (i) −*x*+1, −*y*+1, −*z*+2.
